# Handling of problematic ion chromatograms with the Automated Target Screening (ATS) workflow for unsupervised analysis of high-resolution mass spectrometry data

**DOI:** 10.1007/s00216-024-05245-5

**Published:** 2024-04-01

**Authors:** Georg Braun, Martin Krauss, Stephanie Spahr, Beate I. Escher

**Affiliations:** 1 Department of Cell Toxicology, Helmholtz Centre for Environmental Research – UFZ, Leipzig, Germany; 2 Department of Exposure Science, Helmholtz Centre for Environmental Research – UFZ, Leipzig, Germany; 3 Department of Ecohydrology and Biogeochemistry, Leibniz Institute of Freshwater Ecology and Inland Fisheries (IGB), Berlin, Germany; 4 Environmental Toxicology, Department of Geosciences, Eberhard Karls University Tübingen, Tübingen, Germany

**Keywords:** Mass spectrometry, Target screening, Automation, Workflow, MZmine, TraceFinder

## Abstract

Liquid chromatography (LC) or gas chromatography (GC) coupled to high-resolution mass spectrometry (HRMS) is a versatile analytical method for the analysis of thousands of chemical pollutants that can be found in environmental and biological samples. While the tools for handling such complex datasets have improved, there are still no fully automated workflows for targeted screening analysis. Here we present an R-based workflow that is able to cope with challenging data like noisy ion chromatograms, retention time shifts, and multiple peak patterns. The workflow can be applied to batches of HRMS data recorded after GC with electron ionization (GC-EI) and LC coupled to electrospray ionization in both negative and positive mode (LC-ESIneg/LC-ESIpos) to perform peak annotation and quantitation fully unsupervised. We used Orbitrap HRMS data of surface water extracts to compare the Automated Target Screening (ATS) workflow with data evaluations performed with the vendor software TraceFinder and the established semi-automated analysis workflow in the MZmine software. The ATS approach increased the overall evaluation performance of the peak annotation compared to the established MZmine module without the need for any post-hoc corrections. The overall accuracy increased from 0.80 to 0.86 (LC-ESIpos), from 0.77 to 0.83 (LC-ESIneg), and from 0.67 to 0.76 (GC-EI). The mean average percentage errors for quantification of ATS were around 30% compared to the manual quantification with TraceFinder. The ATS workflow enables time-efficient analysis of GC- and LC-HRMS data and accelerates and improves the applicability of target screening in studies with a large number of analytes and sample sizes without the need for manual intervention.

**Graphical Abstract:**

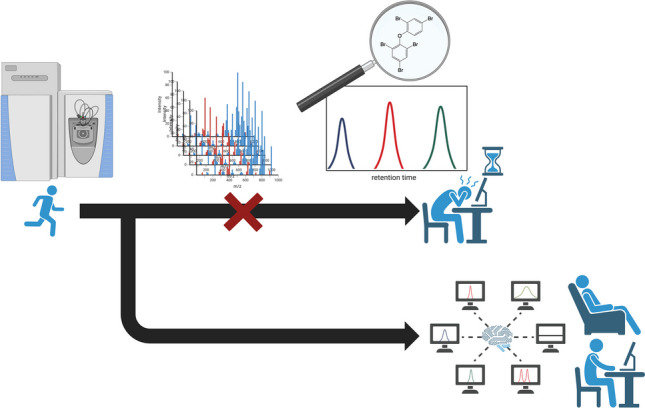

**Supplementary Information:**

The online version contains supplementary material available at 10.1007/s00216-024-05245-5.

## Introduction

The combination of mass spectrometry with liquid or gas chromatography (LC or GC) is widely applied for the detection, identification, and quantification of organic compounds in disciplines such as metabolomics, lipidomics, and human and environmental pollutant monitoring [1, 2]. In particular, the development of very sensitive, high-resolution mass spectrometers (HRMS) with high mass accuracy allows for the detection of hundreds to thousands of compounds in a sample in the same analytical run [3].

In many applications, targeted approaches are of interest, with the aim of identifying and quantifying several hundred chemicals in extracts of complex matrices such as water, sediment, or biota [4–7]. Samples are measured together with pre-selected reference standards to directly annotate peaks, which allows for full quantification within a set calibration range. As the reference spectra are directly available and were measured under identical conditions, the limits of detection and quantification are relatively low and the annotation confidence is high. A major drawback in such target screening is its current lack of automation and throughput. Usually, the data is evaluated using vendor software such as Analyst® (Sciex), MassHunter® (Agilent), or TraceFinder® (Thermo Scientific). These software tools are user-friendly, focus on data visualization and interactivity, and allow corrections of wrong or missing peak annotations and integration by the selected detection algorithms through visual inspection of each peak. However, this approach is very time-consuming and slow in computation, limiting its efficient use to a few analytes and a limited number of samples.

The field of non-target screening (NTS) has become more advanced in terms of automation and efficient data processing, especially when used for LC-HRMS applications [8, 9]. This is due to the nature of NTS to work with as many input information as possible to identify all features of interest. Another common approach that also offers higher throughput and works retrospectively is the suspect screening (SS), where the scan information is trimmed to regions of interest of a suspected list of analytes and their *m/z* and/or retention times. Despite recent improvements in annotation confidence, spectral normalization, and standard-free semi-quantification [10, 11], full quantification is not reliable due to higher annotation uncertainty and depends on the availability of reference spectra for confident annotation, comparison of data from different batches or even instruments.

The need for higher throughput data processing in NTS and SS led to the creation of software such as MZmine, which has been used for partially or fully automatic data processing including smoothing, spectra deconvolution, peak detections, and gap filling with sufficient throughput [12, 13]. In addition to NTS and SS, MZmine can also be used to perform targeted analysis by incorporating user-defined lists of target features and retention times to annotate peaks. The inclusion of reference standards and calibrations improves the annotation confidence of the workflow and can be used for quantification [14]. Due to this increased throughput and the use of algorithms instead of manual data curation, this form of target analysis is often referred to as target screening.

The use of non-targeted peak detection algorithms is rather demanding in terms of computational power and memory due to the aim of identifying all possible features in a dataset. More importantly, these algorithms cannot be easily applied if ion chromatograms have too high background noise or drifts in retention times. Retention time shifts can be caused by the accumulation of impurities on the stationary phase introduced by the sample matrix, by the change of injection volume if bubbles form in syringes of autosamplers, if samples and/or references have not been prepared with the same matrix, and if the sample composition differs strongly from the mobile phases used. Another problem is multiple peak patterns, for example, for closely eluting isomers such as polycyclic aromatic hydrocarbons (PAHs), polychlorinated biphenyls (PCBs), or brominated diphenyl ethers (BDEs) [5]. If detection algorithms designed to identify as many potential features as possible are applied to noisy data, especially in combination with sample-dependent shifts in retention times, this can lead to a high number of wrong or missing peak annotations, even if only focused on a specific set of analytes.

Hence, there is a need for a workflow for HRMS-based target screening that fulfills all of the following criteria: (a) produces confident reference-guided and potentially shift-corrected peak annotation and quantification, (b) is not limited to LC-HRMS measurements but also works for GC-HRMS with comparable quality, (c) can work with problematic (extracted) ion chromatograms that feature different peak shapes, variable noise levels, and signal interferences or multiple peak patterns, and (d) does not depend on the evaluation in licensed vendor programs and still enables comprehensive data visualization.

In this study, we introduce an open-source unsupervised workflow for Automated Target Screening (ATS) of both GC-HRMS and LC-HRMS data and validate its performance against a manual evaluation in TraceFinder (TF) and batch-mode evaluation in MZmine using a real-life data set. Our R-based workflow allows users to automatically analyze and even quantify their targeted high-resolution mass spectrometry data after setting up a working environment and workflow parameters. The workflow allows processing .raw files (Thermo Scientific) or the open MS data format .mzML. Hence, the workflow can be split into ATS_raw and ATS_mzML depending on which input data format was used.

## Materials and methods

The workflow and installation instructions are accessible from the GitHub repository https://github.com/braungeorg/AutomatedTargetScreening. An example environment with scripts, target lists, minified versions of the raw data, and results is accessible on Zenodo via 10.5281/zenodo.10047377. TraceFinder (version 5.1) was used as a reference for both peak annotation comparison and quantification, since it is a fully supervised method. MZmine (version 2.51) was used for comparing the peak annotation performance of ATS to a sophisticated (semi-)automated analysis workflow. MZmine 2.51 was used in this comparison, although a newer version MZmine 3 has been launched during the preparation of the present study. As MZmine 3 still uses the same peak detection algorithms, we consider that the results using the older version are comparable [15].

### Dataset

The samples used in this study were a subset of eight samples (sites A–H) from a study on micropollutants in surface water in Germany, which were filtered over 1.6 µm glass fiber filters to obtain an aqueous phase of freely dissolved and dissolved organic carbon-bound chemicals, and a filter residue that contains all particle-bound chemicals. Organic contaminants were extracted from the aqueous phase using solid-phase extraction (SPE) and from suspended particles using pressurized liquid extraction (PLE). The SPE extracts and PLE extracts were measured with LC-HRMS and GC-HRMS, respectively. The ionization techniques were electrospray ionization (ESI) in both positive and negative modes for LC (LC-ESIpos and LC-ESIneg), as well as electron ionization (EI) for GC (GC-EI). Instrumental analysis for both LC- and GC-HRMS was performed as published by Niu et al. [16]. The details regarding sample extraction and settings of TraceFinder and MZmine can be accessed from Text S1 to Text S3 in Supporting Information S1. The Thermo .raw data files were converted to centroided .mzML using ProteoWizard’s MsConvert tool [17]. Minified versions of the .mzML files of these samples are included in the example files of the R package. These minified versions were created using the RaMS R package (version 1.3.0) [18] and covered only the *m/z* of very few analytes for LC-ESIpos and GC-EI for demonstration purposes.

The list of all analyzed compounds and their IDs and mode of analysis as well as internal standards are accessible from Supporting Information S2, Tables S2-1 and S2-2.

### Workflow

**Fig. 1:**
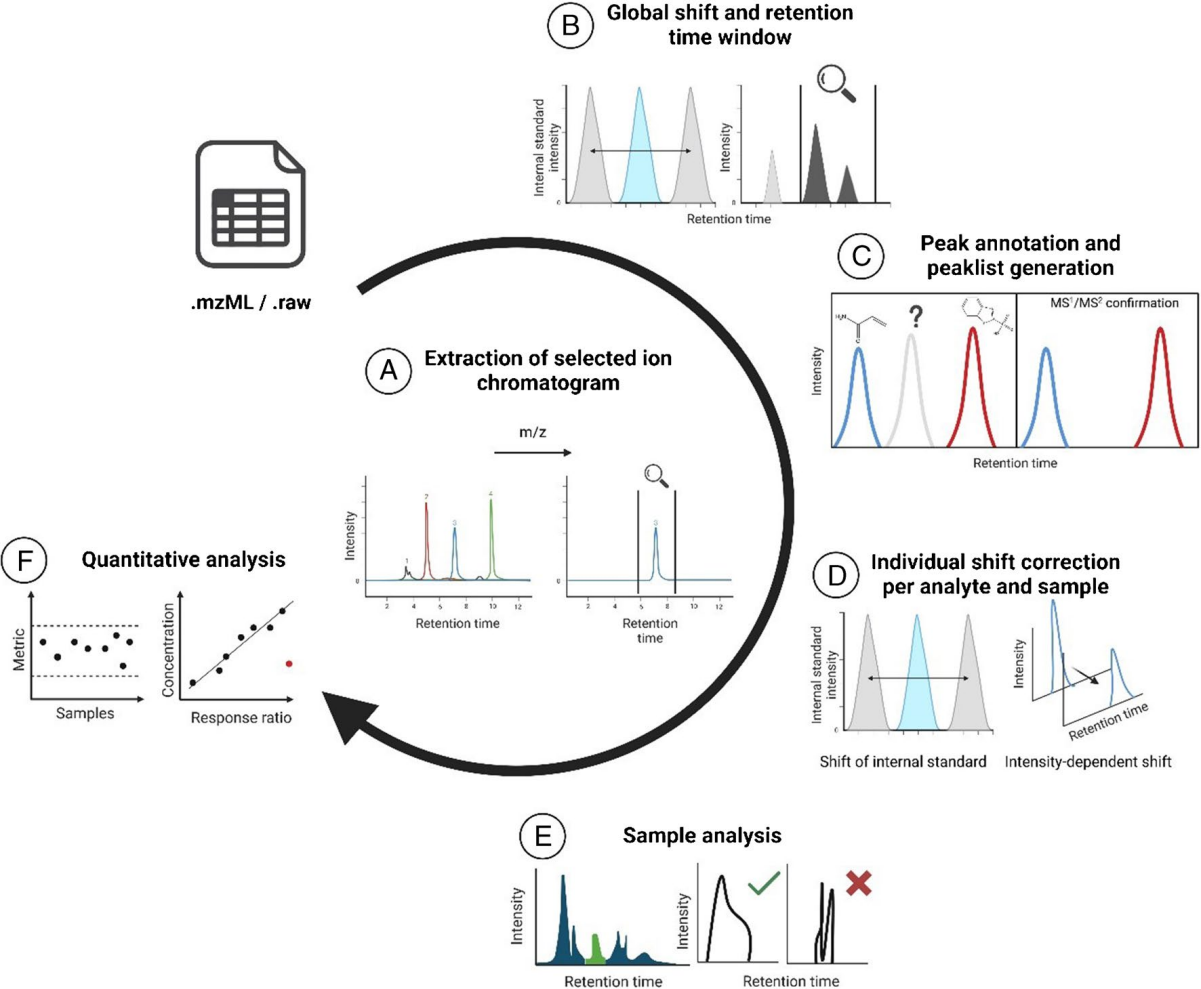
Main functions and steps of the ATS workflow. **A** For each function working with raw data, EICs are generated per *m/z* of interest and processed for further analysis. **B** A global shift for the whole batch is calculated based on internal standards. **C** A peak list is generated using external standards and confirmation ions. **D** In-sample shifts are considered by checking drifts of internal standards as well as intensity-dependent shifts of calibrations. **E** Each sample is analyzed for valid peaks considering shifts and peak shapes of references. **F** Quantitative analysis to obtain metrics like variability of internal standard intensity and recovery or concentrations based on external standards

The ATS R package (R version 4.1.3) [19] can be accessed and installed from GitHub. Installation instructions and details regarding dependencies and required files as well as the setup of the working environment, samples, and variables are included in respective README files in the GitHub repository. An overview with descriptions and impact of the user-defined ATS analysis parameters is also provided in Supporting Information S2, Table S2-3. The workflow consists of six main steps as visualized in Fig. 1. Within the R package, the workflow is split into five main functions. After setting up the working environment, the functions can be called individually, or ran as a batch in the provided Automated_Target_Sreenning_workflow.R script. All functions use extracted ion chromatograms (EICs) and respective symbolic aggregate approximation (SAX) sequences generated using the procedure described in the following. For more detailed information, see Supporting Information S1, Figure S1-1.

#### **Function A: generation of EICs, overall peak identification, and integration**

This function is used as a data import function in all raw data handling functions of the workflow. EICs are generated using the R packages rawrr (version 1.2.0) [20] for .raw and RaMS (version 1.3.0) [18] for .mzML files. A smoothed chromatogram is generated, but only used to increase data density for some of the shape-dependent functions. Quantitative functions such as the calculation of peak heights and areas use the measured intensity values of the EIC without any smoothing. Integrated peak areas are calculated via the trapezoidal rule. The full EIC is described as a symbolic aggregate approximation (SAX) sequence [21, 22] using the seewave package (version 2.2.0) [23] and a selectable alphabet size, which is used to identify and define peaks and their shapes.

An EIC with a broader retention time window is used as a so-called background EIC. This background EIC is used to define the baseline and background noise of the respective narrower EIC of the analyte within a given sample. The timeframe of the background EIC is based on the sample search window and extended by a user-defined factor, in our study 1.5. The initial search window is set by the user and gets trimmed to the reference-guided area of interest for the respective analytes after peak annotation. The use of an extended EIC for background calculation is necessary to avoid artificially elevated baseline and background values, e.g., due to peak fronting or tailing, or if the EIC consists mainly of the analyte peak after trimming to the area of interest, or if co-eluting nearby peaks occur. For baseline calculation in each EIC, shapes like random scatters or highly fluctuating intensities (so-called zigzag patterns) are detected to define which quantiles of the intensities are used. Zigzag is defined as intensity patterns of contiguous sequences of alternating high and close to baseline intensities. Usually, the baseline is defined as the 25% intensity quantile. For more challenging EICs with high-intensity fluctuation, ATS needs to be stricter in defining the baseline values to avoid integrating noise. To detect such noisy EICs, ATS checks several thresholds, including the following: (A) portion of total zigzag patterns of the EIC must be below 25% (defined as zigzag_trigger_threshold, see Table S2-3), (B) the root mean squared error of the background must be below 10%, (C) the ratio of maximum intensity divided by the 25% intensity quantile must be >3 (defined as minimum_background_ratio, see Table S2-3). If one of these criteria is not met, a higher intensity quantile is used to define the elevated baseline due to higher interferences. A quantile threshold of 80% was deemed appropriate in our case study. However, the respective quantiles can be adjusted by the user via the parameters normal_background_quantile and higher_background_quantile (see Table S2-3). The noise is defined as the standard deviation of all baseline-defining intensities. A limit of baseline + 3× noise is used as the in-sample limit of detection (LOD) and baseline + 10× noise as the in-sample limit of quantification (LOQ). These in-sample LOD and LOQ are intensity-based and do not represent the final quantitative LOD or LOQ of the analytes, but aim to improve confident peak annotation.

For peak identification, a local maxima algorithm is used. In brief, the script starts with the highest intensity in a defined retention time window. For each maximum, it follows decreasing intensities until the first non-decreasing intensity value is reached. Based on the data density, which is defined as data points within 0.1 min, a maximum number of non-decreasing intensity values is defined (five times the data density in our study). A peak-related decrease in intensity is considered as continued if within this number of data points a decrease of intensity greater than a defined threshold (>40% in our study) occurs relative to the last intensity value that has been assigned to the peak. If this is not the case, a steady intensity plateau is assumed, and the end of the peak is set at the start of the plateau. Further, an increase of intensity greater than another defined threshold relative to the last assigned intensity (>40% in our study) will trigger another counting of data points. This counting aims to allow small bumps or non-Gaussian deformities to occur within one peak as long as the decrease in intensity continues afterwards. If the increase is however continued for more than a fourth of the data density, the algorithm will be terminated assuming the start of another peak. All these thresholds and factors (intensity_factor_decrease, intensity_factor_increase, density_factor) can be selected by the user (see Table S2-3).

This is repeated for all local maxima within the retention time window. To allow the splitting of merged or intersected peaks, likely cutoffs, defined as the points of contact of the edges of two local maxima, are used as enforced split points. The function also adjusts the respective SAX sequence of the EIC by adjusting the letters to the lowest letter “a” at the start and end retention time of a peak, or at identified necessary split points.

#### **Function B: calculation of global shift**

For the global shift analysis, all listed internal standards (IS) are analyzed and the shifts within all IS-containing samples are calculated. It is assumed that the mean of these shifts represents the global shift, i.e., a probable shift that affects all analytes and is system-dependent, for example, due to the cutting of GC columns. The largest shift is then used as the retention time window to screen for peaks, trying to include all possible scenarios of highly shifted and robust peaks. By default, the retention time window is at least 0.5 min in LC analyses and 0.1 min in GC analyses. Shifts are calculated in relation to the given retention time in the target list of choice.

#### **Function C: peak list annotation and calculation of intensity-dependent shifts**

Within the defined retention time window, all peaks with suitable peak characteristics are listed. Per reference standard, a consensus peak with unified peak characteristics is generated. If defined for an analyte, confirming ions are compared to the quantified ion and used for confirmation and peak selection. If references are used at different concentrations (i.e., calibrations), it is checked whether higher concentrations tend to elute earlier, indicating overload of the column and an intensity-dependent shift. A simple linear regression of intensity and theoretical concentration and a check for unspecific peaks around the aimed retention time are used to reduce falsely identified intensity-dependent peak shifts. Therefore, it is strongly recommended to include a sample with internal standards only and without any quantified reference compounds to identify unspecific and potentially interfering peaks. Besides overload of the system, this step also accounts for changes in the peak shapes of analytes at lower concentrations, which are more likely to be found in samples due to co-elution of other chemicals and matrix effects.

#### **Function D: internal standard analysis**

The shift of the internal standards is calculated for each sample. This shift assumes that compounds are assigned to standards which are likely affected by the same matrix effects like overloads or changes in ionization efficiency, for example, because they are structurally related or at least share similar retention times. Hence, individual shifts of the respective IS indicate a likely shift of the analyte within the given sample(s). If later a valid peak is identified in a sample without shift correction and exactly at the expected retention time, this consideration of the calculated IS-dependent shift will be ignored by the algorithm (assuming a distinct selection of likely peaks using high-resolution instruments). This is relevant if the assigned internal standard is not sharing the same functional groups or physicochemical properties of the analyte.

#### **Function E: sample analysis**

The target screening function can be considered as the main function and uses the retention time derived from the peak list annotation and considers all possible intensity-dependent and/or IS-dependent shifts to define the screening window for the analyte in each sample. Peaks are allowed to have non-optimal shapes such as highly fluctuating intensity patterns (zigzag) or non-Gaussian forms if significant intensities (a priori set range of intensities) occur closely to the expected retention times. Confirming ions are screened separately for their presence and are tested if a detection can be expected. The ratio of quantified ion to confirming ion is used and assumed to be stable with an allowed intensity deviation of 20%. If the expected intensity of the confirming ion exceeds the calculated in-sample LOD, a peak must be found for the confirming ion. If shifts are too far off (defined by pre-defined retention time tolerances and allowed calculated shifts), the peak shapes differ strongly (identified by using a calculated maximum of the MINDIST function to compare SAX sequences of peaks and EICs [21]), the peak consists of very few data points (pre-defined by the user, default is <=7), or an expected confirming ion is missing; the area is integrated but tagged as “CHECK”. These “CHECK” cases are considered negative in a fully unsupervised mode, but can be manually re-evaluated. These “CHECK” cases are similar to the peak status “ESTIMATED” in MZmine.

#### **Function F: quantification of samples**

For quantification, samples have to be grouped into solvent blanks, reference standards, and samples. The reference standards can have the same concentration to be used for recovery calculations, or represent a calibration. Additionally, specific groups of samples such as matrix-matched quality control standards or standards containing references for qualitative peak annotation can be defined. The respective name tags for files and variables are user-defined as instructed on the GitHub repository. The analysis can be performed using responses in the form of peak areas or heights.

First, solvent blanks are screened for responses of analytes. If responses are found in more than one-third of all solvent blanks, the mean response is subtracted from all samples, whereby a system-dependent background is assumed. Only samples that exceed this mean of blanks more than three times are regarded as valid peaks and included for further analyses; otherwise, they are tagged “masked by background.”

If solvent-based reference standards are used but potential matrix effects need to be considered, one can prepare matrix-matched samples spiked with the references and tag those samples as matrix samples. These matrix samples are compared to their solvent-matched counterparts and a matrix correction of the respective analytes is performed for all samples.

If only references with a constant concentration are used, e.g., for calculating recoveries, they are tested for deviation by calculating the interquartile range (IQR) of all references within the batch and checked for outliers. The IQR is chosen due to the lower influence of outliers. If the response of a reference control is outside the calculated accepted intervals, the respective samples are removed from further calculations.

Quantification is performed when the calibration samples are included. Here, a simple linear regression is used in six variants: (1) through origin without weights, (2) through origin with weights = ×, (3) through origin with weights = 1/×, (4) no restrictions, (5) no restrictions with weights = ×, (6) no restrictions with weights = 1/×. The six models were included to increase the likelihood of a good fit for a more diverse set of data with possibly varying trends and patterns. This is in particular relevant in the process of the autotrimming of imperfect linear calibration curves, for example, by removing bent regression trends or constant background signals, which cover responses at low concentrations. The regressions are autonomously optimized against the coefficient of determination (which needs to be at least a pre-defined minimum, default is 0.95) and the median error of quantification (which should be lower than a pre-defined value, default is < 30%). The error for quantification was calculated according to Eq. (1).

1 $$\mathrm{Error}\;\mathrm{quantififcation}\;(\%)=\mathrm{median}\;\left(\left|\left(\frac{{\mathrm C}_{\mathrm{actual}}-{\mathrm C}_{\mathrm{predicted}}}{{\mathrm C}_{\mathrm{actual}}}\right)\ast100\right|\right)$$

Concentration value(s) with the highest leverage are identified using Cook’s distance. Such high-leverage concentrations are removed if their exclusion results in a decrease in the error of quantification and an increase in the coefficient of determination. This step is repeated as long as the optimization parameters improve and the calibration curve consists of enough data points (default at least 4). Cutoffs were defined for the *y*-axis (lowest concentration suitable for quantification, “limit of quantification”) and the *x*-axis (lowest response ratio within the linear range). Hence, the regressions were automatically trimmed to a suitable linear range for quantification. The final regression and concentration range is selected out of all variants which meet the pre-defined criteria by going from highest to lowest impact: (1) lowest error of quantification as calculated by Eqs. (1), (2) highest coverage of the concentration range, (3) highest coefficient of determination.

Per analyte, the ratio of the response ratios from quantified samples and the median response ratio of all valid reference samples which translates to the 100% equivalent are used to calculate the recovery.

For each of the targeted compounds, the output consists of the responses of all samples, the response ratios of all samples, the quantified concentrations of all samples, the quantified values of all samples partially excluded (masked by background, above or below calibration, invalid references, “CHECK” etc.), the consensus peak list of all analytes, and apex retention times of all sample-analyte pairs. Further, a heatmap consisting of TRUE/FALSE/masked by background cases serving as a qualitative overview is also generated.

### Workflow comparison

#### **Peak annotation**

Compounds were selected for peak annotation performance comparison if they had at least one annotated response in one of the three evaluation workflows TraceFinder, ATS, or MZmine. Hence, the total number *n* of peaks for all methods per mode of analysis consisted of the number of selected chemicals times the number of all samples. The peak annotation was compared by calculating the fractions of false positives (FP), false negatives (FN), true positives (TP), and true negatives (TN) of all observations *n* for MZmine and ATS compared to the manual evaluation in TraceFinder. For ATS, both ATS_mzML and ATS_raw were used for comparison. Only features with the “DETECTED” peak status were included in MZmine. For evaluation, the parameters sensitivity, specificity, and accuracy were calculated according to Eqs. (2)–(4) per mode of analysis (LC-ESIpos, LC-ESIneg, GC-EI).

2 $$\mathrm{Sensitivity } = \frac{\mathrm{TP}}{\mathrm{TP+FN}}$$

3 $$\mathrm{Specificity } = \frac{\mathrm{TN}}{\mathrm{TN+FP}}$$

4 $${\mathrm{A}}\mathrm{ccuracy = }\frac{\mathrm{TN+TP}}{\mathrm{TN+FP+TP+FN}}= \frac{\mathrm{TN+TP}}{n}$$

#### **Quantification accuracy**

The accuracy of quantified results was checked by comparing pairs of valid calibrations, determined by having a coefficient of determination (*R*^2^) > 0.95 and an error of quantification <20%. The data was visualized as a Bland-Altman plot using an accepted deviation of 1.96^.^ times the standard deviation. Here, the data is compared as a result of method A (e.g., TraceFinder) and a result of method B (e.g., ATS), and respective differences are plotted against the mean of the data pairs. This visualization enables a direct comparison of the results generated by different methods and the identification of systematic errors and trends. A mean absolute percentage error (MAPE) was calculated according to Eq. (5). Here, the concentrations per analyte i from TraceFinder (C_i,TF_) and ATS (C_i,ATS_) for all *n* analytes were used.

5 $$\mathrm{MAPE}\;(\%)=\frac{\sum_{\mathrm n}^{\mathrm i}\left[{\displaystyle\frac{\left|{\mathrm C}_{\mathrm i,\mathrm{TF}}-{\mathrm C}_{\mathrm i,\mathrm{ATS}}\right|}{{\mathrm C}_{\mathrm i,\mathrm{TF}}}}\ast100\right]}n$$

These analyses were performed for each instrument mode (LC-ESIpos, LC-ESIneg, GC-EI).

## Results

The lists of all detected peaks per workflow as well as quantified concentrations for the 680 analyzed compounds are listed in Supporting Information S2, Text S5, Tables S2-4 and S2-5. Further, a summary of the number of detected compounds per mode of analysis and sampling site is given in Supporting Information S1, Table S1-3. The highest number of chemicals was identified in TF while ATS and MZmine have a lower number but similar patterns of detected chemicals. LC-ESIpos was the instrument mode which had the most detects but also the highest number of target analytes (see Supporting Information S2, Table S2-1). A heatmap of the concentrations of 159 chemicals from ATS and TF (Supporting Information S1, Figure S1-2) shows that ATS has a lower overall detection density compared to TF, but similar patterns in detected chemicals for the samples. For example, the sampling sites A and H can be identified in both analyses as the sites with the most detected chemicals and are accordingly the most polluted. The average runtime of ATS can be summarized as 0.3 min per analyte and 2.3 min per sample file (see Table S1-4).

### Peak annotation

The comparison of the peak annotation (see Supporting Information S2, Table S2-4) via ATS_raw, ATS_mzML, and MZmine with selected key figures is listed in Table 1. TF was used as a reference. For the MZmine analysis of the GC dataset, two sets of retention times were used, (i) a list with retention times as used by ATS with shifts in the range of −1.1 to −0.9 min simulating the case of shifted and not optimized analysis conditions and (ii) the same list of analytes but with exact, shift-corrected retention times as used with TF.

**Table 1:** Results of the peak annotation comparison of ATS and MZmine

Instrument mode	Workflow	TP	TN	FP	FN	Sensitivity	Specificity	Accuracy
LC-ESIpos	ATS_raw	611	1029	142	130	0.82	0.88	0.86
	ATS_mzML	516	1086	87	223	0.70	0.93	0.84
	MZmine	497	1027	131	257	0.66	0.89	0.80
LC-ESIneg	ATS_raw	203	334	83	28	0.88	0.80	0.83
	ATS_mzML	172	356	65	58	0.74	0.85	0.81
	MZmine	184	317	75	72	0.72	0.81	0.77
GC-EI	ATS_raw	429	165	69	121	0.78	0.71	0.76
	ATS_mzML	382	171	63	168	0.69	0.73	0.71
	MZmine	36	209	172	367	0.09	0.54	0.31
	MZmine_sc*	365	163	71	185	0.66	0.70	0.67

^*^sc shift-corrected, *TP* true positives, *TN* true negatives, *FP* false positives, *FN* false negatives. TraceFinder was used as reference. Sensitivity, specificity, and accuracy calculated according to Eqs. (2) to (4)

Overall, ATS_raw was the best-performing workflow in terms of accuracy in all three instrument modes, followed by ATS_mzML (see Table 1). The greatest improvement was observed for the sensitivity, which increased around 1.3-fold if ATS_raw is used compared to MZmine. The results were rather similar for all three workflows for the GC-EI dataset. However, this could only be achieved for the shift-corrected dataset for MZmine, where the retention times were adjusted a priori to exactly match the correct times as used in TF. ATS was able to achieve these results while retention times were shifted per compound in a random range of −1.1 to −0.9 min.

### **Quantification accuracy**

**Fig. 2:**
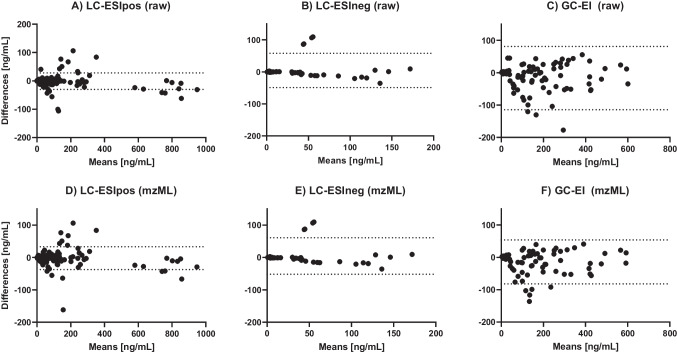
ATS compared to TraceFinder (TF) data. Bland-Altman plots of the three instrument modes LC-ESIpos, LC-ESIneg, GC-EI. **A** *n* = 378, upper threshold = 28 ng/mL, lower threshold = −30 ng/mL. **B** *n* = 54, upper threshold = 58 ng/mL, lower threshold = −49 ng/mL. **C** *n* = 98, upper threshold = 81 ng/mL, lower threshold = −114 ng/mL. **D** *n* = 295, upper threshold = 33 ng/mL, lower threshold = −37 ng/mL. **E** *n* = 49, upper threshold = 61 ng/mL, lower threshold = −52 ng/mL. **F** *n* = 84, upper threshold = 54 ng/mL, lower threshold = −82 ng/mL. The thresholds are visualized as dashed lines and are 1.96 times the standard deviation

The concentrations as listed in Supporting Information S2, Table S2-5, were compared for the two ATS workflows and TF. The comparison for the respective instrument modes is illustrated as Bland-Altman plots in Fig. 2.

**Fig. 3:**
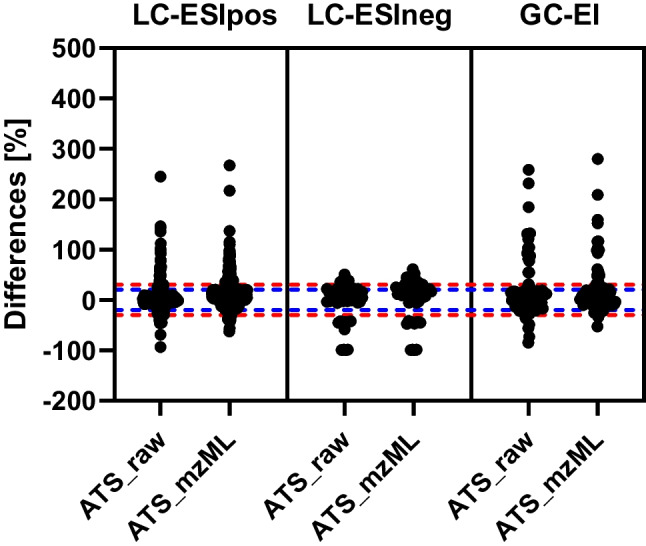
Percentual differences of ATS compared to TraceFinder (TF) data per instrument mode. LC-ESIpos: ATS_raw (*n* = 378), ATS_mzML (*n* = 295). LC-ESIneg: ATS_raw (*n* = 54), ATS_mzML (*n* = 49). GC-EI: ATS_raw (*n* = 98), ATS_mzML (*n* = 84). Dotted lines are 20% deviation (blue) and 30% deviation (red)

The concentration differences were transformed into percentual differences based on the concentrations quantified in TF. The respective plots of the percentual deviations are illustrated in Fig. 3. Considering the percentual deviations, the distributions accumulated in the acceptable ranges of 20–30% (see Fig. 3). LC-ESIneg had a rather even distribution of over- and underestimated values. LC-ESIpos and GC-EI were skewed towards overestimation by ATS. The MAPE values per instrument mode calculated with Eq. (5) were 16% (*n* = 370) and 20% (*n* = 284) for LC-ESIpos, 24% (*n* = 54) and 30% (*n* = 49) for LC-ESIneg, and 34% (*n* = 98) and 30% (*n* = 84) for GC-EI for ATS_raw and ATS_mzML, respectively.

## Discussion

The primary factors considered in the workflow comparison were the performance of ATS in terms of peak annotation and quantification.

### Peak annotation

ATS increased overall performance and accuracy compared to MZmine (see Table 1). While some key numbers like sensitivity and specificity or even accuracy are in a similar range for ATS and MZmine (see Table 1), one has to consider that ATS was using a target feature list which was randomly shifted in the range of −1.1 and −0.9 min while MZmine was only performing well if retention times were exactly as observed for the datasets. Hence, one of the major advantages when using ATS is the unsupervised aspect by making manual intervention and data pre- and post-processing redundant while also increasing accuracy. This advantage of ATS is also emphasized considering the fast runtime of 0.3 min per analyte and 2.3 min per sample file (see Table S1-4), which are unlikely to be achieved in any (semi-)supervised evaluation.

One important aspect of ATS was the processing of the local in-sample background of the EIC and defining respective intensity thresholds of confidence for identifying valid peaks and excluding noise and interfering peaks. This also increased the confidence of peak picking at lower intensity values and noisier baselines.

ATS excels particularly in cases of multiple peaks where manual evaluation was often necessary before. This includes shifted peaks and in-batch changes of retention times, but more specifically cases of expected peak patterns. For MZmine annotation, retention times of the compound list have to be adjusted prior to annotation in another software (e.g., R or MS Excel), and in TF all expected retention times can be adjusted after visual inspection globally in the method or in relation to internal standards serving as retention time reference. Still, this does not ensure the correct assignment of shifted peaks and does not include compound-specific problems like intensity-dependent shifts. Here, ATS helps to save time by making post-processing mostly redundant and avoiding pre-processing steps by allowing the use of target feature lists without adjusted retention times, as long as the retention times were once correctly assigned relative to each other within the target list. This was clearly demonstrated for the GC-EI dataset, which performed similarly for the shift-corrected MZmine dataset and the artificially shifted ATS datasets (see Table 1).

Examples are the fragrances galaxolide and tonalide, which elute very closely in our GC analysis, have the same *m/z* of 243.1743 and show very different ion intensities at the same concentration. In this case, MZmine lists and assigns both peaks to both compounds in the final feature list, while ATS was able to split the peaks and assign the chemicals correctly (see Supporting Information S1, Figure S1-3A). Another example is Cyclopenta(cd)pyren−3,4−H−one, which forms three peaks by default. One strategy would be to assign three different features (peaks 1–3) and evaluate them independently in MZmine or manually integrate the three peaks together in TF. ATS was able to assign all peaks to this compound and adjust acceptable retention time ranges accordingly (see Supporting Information S1, Figure S1-3B). Examples of cases where shifts could interfere with correct detection are the isomers benzo[a]pyrene and benzo[e]pyrene, both of which have *m/z* 252.0933 (see Supporting Information S1, Figure S1-3C). ATS correctly identified and assigned the respective peak of the EIC, whereas MZmine was only able to do so if the retention times had been accurately shift-corrected and small RT windows in advance. Otherwise, the peak finder of MZmine detects two peaks which have a similar peak pattern but the wrong retention time. This shows that shift corrections and selection of small RT windows are crucial if EICs have multiple peaks by default and cannot simply be solved by increasing the retention time tolerances of search windows. Common other examples where this becomes relevant due to co-eluting isomers and shared *m/z* are other PAHs, BDEs, and PCBs. ATS can help to improve and accelerate the data analysis for such chemical groups by automatically handling and assigning the peaks correctly without the need for supervision, even with drifted retention times.

ATS_mzML tended to be less sensitive compared to ATS_raw, mainly due to the lower data density and sensitivity of the imported .mzML files compared to the .raw files. When extracted by packages such as RaMS, EICs from .mzML files consist of significantly fewer data points due to loss during the import. Therefore, it was not possible to recreate the same EICs in detail as with the actual raw data of the .raw files. Furthermore, the extraction of MS^2^ spectra was mostly not possible with mzML-converted files. The solution would be to identify better R packages to generate and process EICs from mzML data, which are compatible with the workflow. Known packages for data extraction like MSnBase [24] could not be implemented since the analysis could not be parallelized.

The use of confirmation ions is important for confident peak annotation and holds one of the highest priorities for peak annotation within the workflow. However, it is also a limiting factor since confirming ions are typically lower in intensities than the target ions and often not detected at lower concentrations. The ATS workflow copes with this by testing whether a confirmation ion can be expected based on intensity ratios and noise levels, and tags identified peaks without present confirming ions. However, due to the loss of data points and sensitivity in the mzML processing, those confirmation ions are sometimes lost, even at the MS^1^ level. Still, ATS_mzML was well-performing and can be considered a valid tool for mzML files, which will be optimized in the future. There are new emerging algorithms and packages like qBinning, which tend to improve the quality of EICs [25].

Overall, the limits of the ATS are mainly peak patterns of signals split into several narrow and highly fluctuating spikes of intensity (zigzag peaks) (see Supporting Information S1, Figure S1-4A and B) or high baseline values (see Supporting Information S1, Figure S1-4C). For the peaks with highly fluctuating intensity values, two functions with opposite aims are relevant. One is to include bad peak shapes which occur exactly at aimed retention times within the retention time window of the identified peak. The other function is relevant to split merged peaks.

The most important user-defined parameter is the minimum_cutoff_intensity_factor (see Table S2-3), which is used to set an intensity threshold based on the height of the highest reference compound. This threshold defines which intensity level is facing the peak-splitting function (above the threshold) and where fluctuating intensity patterns are fully allowed (below the threshold). In our study, this factor was 0.001 meaning that peaks with a rather high intensity and, if linearity is applied, above 1 ng/mL (LC) and 0.5 ng/mL (GC) were split. This means that several samples, where zigzag patterns occurred at even higher concentrations/intensities, were split and hence no correct peak could be identified (see Supporting Information S1, Figure S1-4A). On the other hand, since the full retention time window of the peak identified in references is used and ATS detects intensities above the in-sample thresholds for identification, some peaks are falsely identified by excluding the peak shape as a quality criterion. One option would be the use of MINDIST, which is a quantitative measure of the similarity of SAX sequences of peaks annotated from references and identified sample peaks. This is an option in ATS via the parameter use.MINDIST (see Table S2-3) which was not used in this study. Hence, the results for ATS are dependent on selected workflow parameters and one must define in advance whether it is more important for the analysis to include more possible features or to be stricter in exclusion of less confident features. The parameters most influential on the results are tagged with a high impact in Table S2-3 and testing altered values and combinations can become necessary for different studies and will be part of the optimization of ATS.

### **Quantification accuracy**

The majority of samples for all instrument modes were quantified with a deviation within a percentage error of 100% (see Fig. 3). The MAPE values can be summarized to be around 20–30% compared to the evaluation in TraceFinder. This margin of error is also considered acceptable for the calculation of chemical recoveries in extraction methods [26]. Further, one has to consider that the identified chemicals of this study occur at concentrations at the lower end of the quantified range, as visible in the Bland-Altman plots (see Fig. 2), and hence, high relative deviations in calculated concentrations close to detection limits are not as critical in an absolute point of view. An important feature of ATS is the checking of solvent blanks for significantly frequent analyte intensities. This avoids the incorporation of system-dependent contaminations such as carry-over or material contamination references as valid detects in the final evaluation and helps tag problematic analytes.

Still, if quantification is considered highly important and a mean deviation of around 30% is considered too high, there is potential for further optimization by extending the simple linear regression model to other variants like quadratic calibration [27], splitting the calibration into multiple linear subsections, or manually trimming calibration curves instead of automatically removing calibration points. Also, one could narrow down the range of concentrations and either focus on higher or lower concentrations to increase the accuracy. This study had a rather large concentration range from 0.1 to 1000 ng/mL (LC) or 0.5 to 500 ng/mL (GC), and hence, the lower concentrations naturally tend to have higher relative errors if calibrations are used for the whole concentration range.

ATS is specifically designed for target screening, where the focus lies on dealing with the complexity of the dataset and the handling of hundreds of chemicals in up to hundreds of samples. Its advantage is the in-workflow quantification of many analytes by automatically defining respective quantifiable ranges without the need for additional data processing steps, and is by definition not aiming for the most accurate quantification of distinct analytes.

## Conclusion

In summary, with ATS we have introduced an analysis workflow which can be used to process HRMS data with a highly reduced need for intervention by the user and fast processing speed. ATS is able to increase the overall peak annotation accuracy and outperforms sophisticated workflows like MZmine for challenging scenarios like shifted retention times or co-eluting analytes. The ATS workflow further allows for in-process quantitation with automated determination of linear range and limits of quantitation and detection with calculated concentrations deviating around 30% compared to manual quantification in TF. In our laboratory, we routinely apply the ATS workflow for studies that have both large numbers of samples and analytes, including water quality testing and human biomonitoring samples, where we need fast, automated, and reliable tools to process such large numbers of samples in both GC and LC to cover a wide range of analytes.

### Supplementary Information

Below is the link to the electronic supplementary material.

Supplementary file1 (DOCX 3720 KB)

Supplementary file2 (XLSX 227 KB)
