# Supplementary material for: Handling of problematic ion chromatograms with the Automated Target Screening (ATS) workflow for unsupervised analysis of high-resolution mass spectrometry data
Source: Anal Bioanal Chem. 2024 Apr 1;416(12):2983–93. doi: 10.1007/s00216-024-05245-5 (PMC11045623; doi:10.1007/s00216-024-05245-5)
Supplement: Supplementary file 1 — Supplementary file1 (DOCX 3720 KB) [file 216_2024_5245_MOESM1_ESM.docx]

# Supporting Information S1

# Handling of problematic ion chromatograms within the Automated Target Screening (ATS) workflow for unsupervised analysis of high-resolution mass spectrometry data

Georg Braun*^1^, Martin Krauss^2^, Stephanie Spahr^3^, and Beate I. Escher^1,4^

^1^Department of Cell Toxicology, Helmholtz Centre for Environmental Research – UFZ, Leipzig, Germany

^2^Department of Exposure Science, Helmholtz Centre for Environmental Research – UFZ, Leipzig, Germany

^3^Department of Ecohydrology and Biogeochemistry, Leibniz Institute of Freshwater Ecology and Inland Fisheries (IGB), Berlin, Germany

^4^Environmental Toxicology, Department of Geosciences, Eberhard Karls University Tübingen, Tübingen, Germany

*Corresponding author: Georg Braun, E-Mail: Georg.braun@ufz.de

**Table of content**

**Tables:**

Table S1-1: TraceFinder settings 2

Table S1-2: MZmine settings 2

Table S1-3: Summary of the number N of detected compounds per mode of analysis and sampling site for TraceFinder, MZmine, and ATS. 5

Table S1-4: Runtime comparison of ATS for all samples 9

**Figures:**

Figure S1-1: Extracted ion chromatogram processing steps. 4

Figure S1-2: Heatmap of the concentrations in log ng/mL for TraceFinder and ATS and all sampling sites. 6

Figure S1-3: Examples of cases where ATS is outperforming MZmine. 7

Figure S1-4: Examples of problematic compounds in ATS. 8

**Text S1. Sample preparation.**

Water samples were filtered with glass fiber filters (GFF, pore size 1.6 μm, Fisherbrand, Fisher Scientific) to separate the water phase from the particles. Solid-phase extraction (SPE) was performed to extract organic contaminants from the water phase. Oasis HLB cartridges (6 cc, 500 mg sorbent, Waters, MA, USA) were conditioned with 10 mL ethyl acetate (EtAc), 10 mL methanol (MeOH), and 10 mL HPLC-grade water. Cartridges were loaded with 1 L of sample using a vacuum pump. SPE blanks were prepared accordingly with HPLC-grade water. The cartridges were dried under vacuum and eluted with 10 mL MeOH and 10 mL EtAc. The extracts were evaporated to dryness with a gentle N_2_ stream, and redissolved in MeOH to achieve an enrichment factor of 1000.

The particle-loaded GFF-filters were freeze-dried and subsequently extracted using accelerated solvent extraction (ASE 350, Dionex, Thermo Fisher Scientific, Waltham, MA, USA). Filtration of 3 L of aqueous sample resulted in 7 – 10 particle-loaded filters, which were jointly extracted in one 34 mL stainless steel extraction cell. Two 1.6 μm GFF filters were added at the bottom of each cell. EtAc and acetone were used with ratio 1 : 1 as extraction solvents under two cycles (5 min preheat, 5 min heat, and 10 min static) at 100°C and 100 bar. Afterwards, the extracts were evaporated to dryness, redissolved in 500 μL dichloromethane (DCM) and subjected to a clean-up procedure as described in Niu, Henneberger, Huchthausen, Krauss, Ogefere and Escher [1] Briefly, silica gel cartridges (Chromabond Flash, Macherey-Nagel, Düren, Germany) were conditioned with DCM. Subsequently, the ASE extracts were loaded onto the cartridges and eluted with DCM. The DCM extracts were evaporated to dryness and redissolved in 500 μL of EtAC for GC-HRMS analysis. Blanks were obtained by extracting GFF filters or filters with hydromatrix and processed in the same way as the samples.

**Text S2. TraceFinder settings.**

The settings used for TraceFinder, version 5.1, build 203, are listed in Table S1-1.

Table S1-1: TraceFinder settings

| Parameter | Value |
| --- | --- |
| Mass tolerance | 7 ppm |
| Background subtraction | FALSE |
| Detection Algorithm | ICIS |
| Peak detection strategy | Nearest RT |
| Peak threshold type | Height |
| Threshold | 1 |
| Smoothing | 5 |
| Extraction window | 4 min |
| Area noise factor | 3 |
| Peak noise factor | 10 |
| Baseline window | 100 |
| Min peak height (S/N) | 10 |
| Noise method | Incos |
| Min peak width | 8 |
| Multiplet resolution | 8 |
| Area tail extension | 5 |
| Area scan window | 0 |

Quantitation was performed by using linear regression with weights 1/x and no enforced intercept. The acquisition list consisted of the compounds listed in Supporting Information S2, Table S2-1.

**Text S3. MZmine settings.**

MZmine version 2.51 was run in batch mode and the following batch queue and parameters as listed in Table S1-2 were used. The reference database consisted of the target list as defined by the compounds of Supporting Information S2, Table S2-1.

Table S1-2: MZmine settings

| Function | Parameter | Unit (GC) | Unit (LC) |
| --- | --- | --- | --- |
| Mass detection | MS level | 1 | 1 |
|  | Mass detector | Centroid | Centroid |
|  | Noise level | 5.0E3 | 5.0E3 |
| ADAP Chromatogram builder | MS level | 1 | 1 |
|  | Min group size in # of scans | 10 | 8 |
|  | Group intensity threshold | 8.0E3 | 1.0E4 |
|  | Min highest intensity | 5.0E3 | 5.0E3 |
|  | m/z tolerance | 0.001 – 7.0 ppm | 0.001 – 7.0 ppm |
| Smoothing | Filter width | 7 | 7 |
| Chromatogram deconvolution | Algorithm | Local minimum search | Local minimum search |
|  | m/z center calculation | MEDIAN | MEDIAN |
|  | Chromatographic threshold | 30 % | 60% |
|  | Search minimum | 0.02 min | 0.1 min |
|  | Minimum relative height | 0.1% | 30 % |
|  | Minimum absolute height | 5.0E4 | 5.0E4 |
|  | Min ratio of peak top/edge | 2.1 | 2.3 |
|  | Peak duration range | 0.02 – 1.5 min | 0.1 – 5.0 min |
| Sort feature list | - | -- | - |
| Join aligner | m/z tolerance | 0.001 – 7.0 ppm | 0.001 – 7.0 ppm |
|  | Weight for m/z | 70% | 70% |
|  | Retention time tolerance | 0.15 min | 0.3 min |
|  | Weight for RT | 30 | 30 |
| Peak finder | Intensity tolerance | 30 % | 30 % |
|  | m/z tolerance | 0.001 – 7.0 ppm | 0.001 – 7.0 ppm |
|  | Retention time tolerance | 0.1 | 0.15 |
| Custom database search | Database file | Table S2-1 | Table S2-1 |
|  | m/z tolerance | 0.001 – 7 ppm | 0.001 – 7 ppm |
|  | Retention time tolerance | 0.2 min | 0.6 min |

The feature lists were exported and only features with the “DETECTED” status were processed as identified peaks by MZmine for the comparison.

**Text S4. Extracted Ion Chromatogram processing.**

The generated extracted ion chromatograms (EICs) are handled by six major steps which are visualized in Figure S1-1.


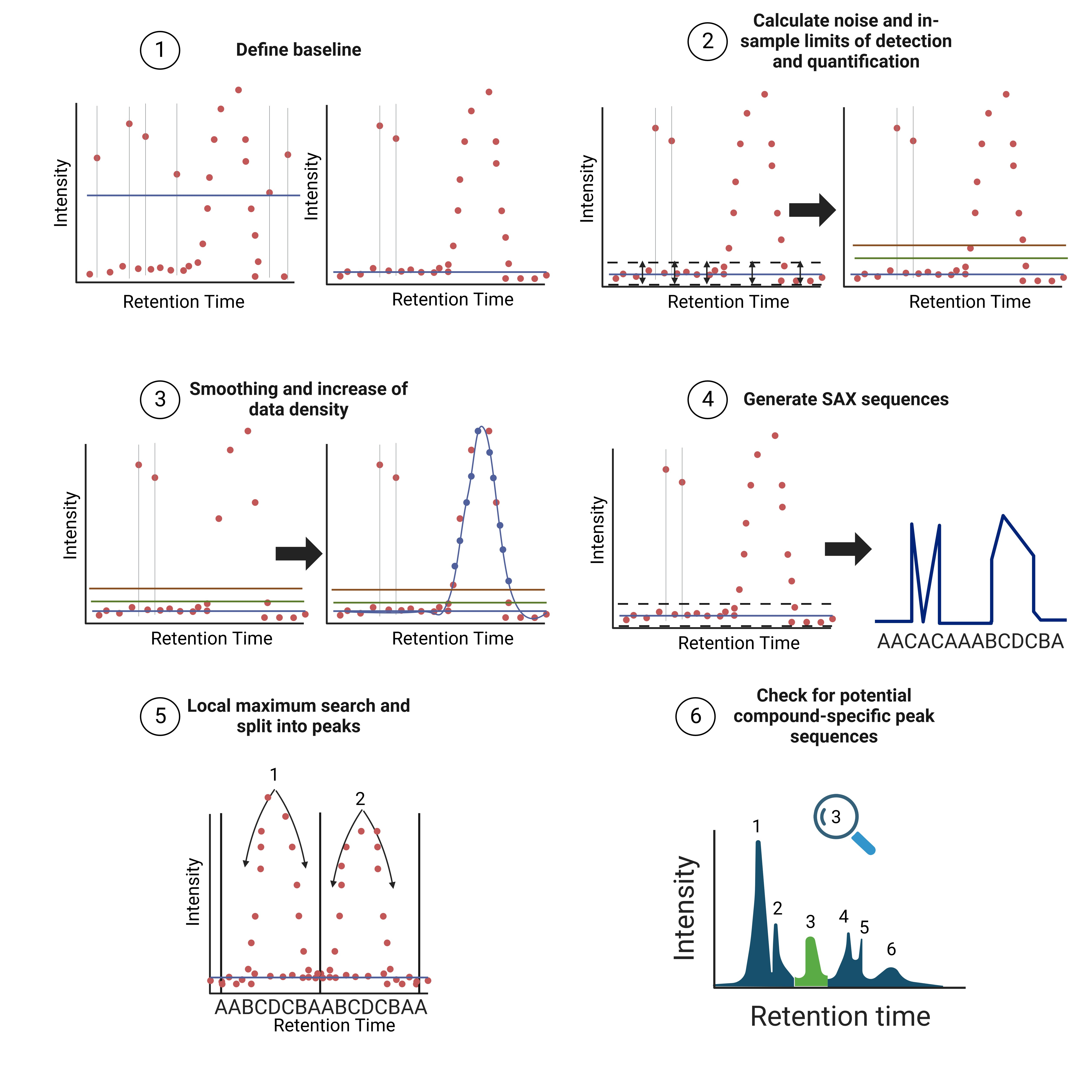


Figure S1-1: Extracted ion chromatogram processing steps.

SAX = symbolic aggregate approximation

First, a baseline is defined dependent on surrounding intensities (flat, highly fluctuating patterns). Second, in-sample limits of detection and quantitation are calculated based on the noise, defined by the standard deviation of values defining the baseline. Third, the EIC is smoothed to increase data density. Fourth, the whole EIC is transformed into symbolic aggregate approximation (SAX) sequences based on intensity and with defined alphabet size. These SAX sequences allow the decoding of varying peak shapes into clear sequences which can be compared or used for identification [2, 3]. Fifth, a local maximum algorithm is defining peaks and the EIC is split into sections of peaks. Sixth, when defining the peaks of interest and pre-defined by the user, only peaks are selected which occur as expected by potential sequences of multiple peaks, for example double peaks or isobaric analytes with similar structures within the search window. This step is only applied when generating the reference peaklist.

**Text S5. Results of the sampling sites with ATS, MZmine and TraceFinder.**

Of all analyzed chemicals (see Supporting Information S2, Table S2-1), the respective detected cases of Supporting Information S2, Table S2-4 are summarized per mode of analysis and sampling site in Table S1-3.

Table S1-3: Summary of the number N of detected compounds per mode of analysis and sampling site for TraceFinder, MZmine, and ATS.

| TraceFinder | | | | | | | | |
| --- | --- | --- | --- | --- | --- | --- | --- | --- |
| **N detected** | **Site_A** | **Site_B** | **Site_C** | **Site_D** | **Site_E** | **Site_F** | **Site_G** | **Site_H** |
| **LC-ESIpos** | 149 | 147 | 496 | 87 | 496 | 86 | 84 | 137 |
| **LC-ESIneg** | 40 | 42 | 36 | 35 | 35 | 35 | 36 | 43 |
| **GC-EI** | 44 | 45 | 45 | 45 | 46 | 46 | 46 | 47 |
| **Total** | 233 | 234 | 577 | 167 | 577 | 167 | 166 | 227 |
| **MZmine_sc** | | | | | | | | |
| **N detected** | **Site_A** | **Site_B** | **Site_C** | **Site_D** | **Site_E** | **Site_F** | **Site_G** | **Site_H** |
| **LC-ESIpos** | 110 | 121 | 80 | 72 | 77 | 79 | 78 | 114 |
| **LC-ESIneg** | 30 | 29 | 26 | 24 | 26 | 27 | 28 | 30 |
| **GC-EI** | 48 | 44 | 46 | 45 | 44 | 42 | 44 | 46 |
| **Total** | 188 | 194 | 152 | 141 | 147 | 148 | 150 | 190 |
| **MZmine** | | | | | | | | |
| **N detected** | **Site_A** | **Site_B** | **Site_C** | **Site_D** | **Site_E** | **Site_F** | **Site_G** | **Site_H** |
| **LC-ESIpos** | 110 | 121 | 80 | 72 | 77 | 79 | 78 | 114 |
| **LC-ESIneg** | 30 | 29 | 26 | 24 | 26 | 27 | 28 | 30 |
| **GC-EI** | 22 | 21 | 25 | 23 | 25 | 19 | 23 | 25 |
| **Total** | 162 | 171 | 131 | 119 | 128 | 125 | 129 | 169 |
| **ATS_raw** | | | | | | | | |
| **N detected** | **Site_A** | **Site_B** | **Site_C** | **Site_D** | **Site_E** | **Site_F** | **Site_G** | **Site_H** |
| **LC-ESIpos** | 145 | 142 | 97 | 81 | 87 | 94 | 88 | 142 |
| **LC-ESIneg** | 29 | 29 | 25 | 26 | 25 | 27 | 25 | 30 |
| **GC-EI** | 46 | 47 | 44 | 47 | 45 | 48 | 47 | 52 |
| **Total** | 220 | 218 | 166 | 154 | 157 | 169 | 160 | 224 |
| **ATS_mzML** | | | | | | | | |
| **N detected** | **Site_A** | **Site_B** | **Site_C** | **Site_D** | **Site_E** | **Site_F** | **Site_G** | **Site_H** |
| **LC-ESIpos** | 118 | 118 | 70 | 73 | 74 | 77 | 72 | 107 |
| **LC-ESIneg** | 34 | 32 | 24 | 27 | 27 | 27 | 25 | 32 |
| **GC-EI** | 42 | 44 | 44 | 46 | 44 | 45 | 46 | 50 |
| **Total** | 194 | 194 | 138 | 146 | 145 | 149 | 143 | 189 |

The concentrations in ng/mL of chemicals identified in the sampling sites via TraceFinder and ATS are listed in Supporting Information S2, Table S2-5 and are visualized in Figure S1-2.

Figure S1-2: Heatmap of the concentrations in log ng/mL for TraceFinder and ATS and all sampling sites.

n = 159 chemicals used for the quantitative analysis.

**Text S6. Examples of cases where ATS outperforms MZmine.**


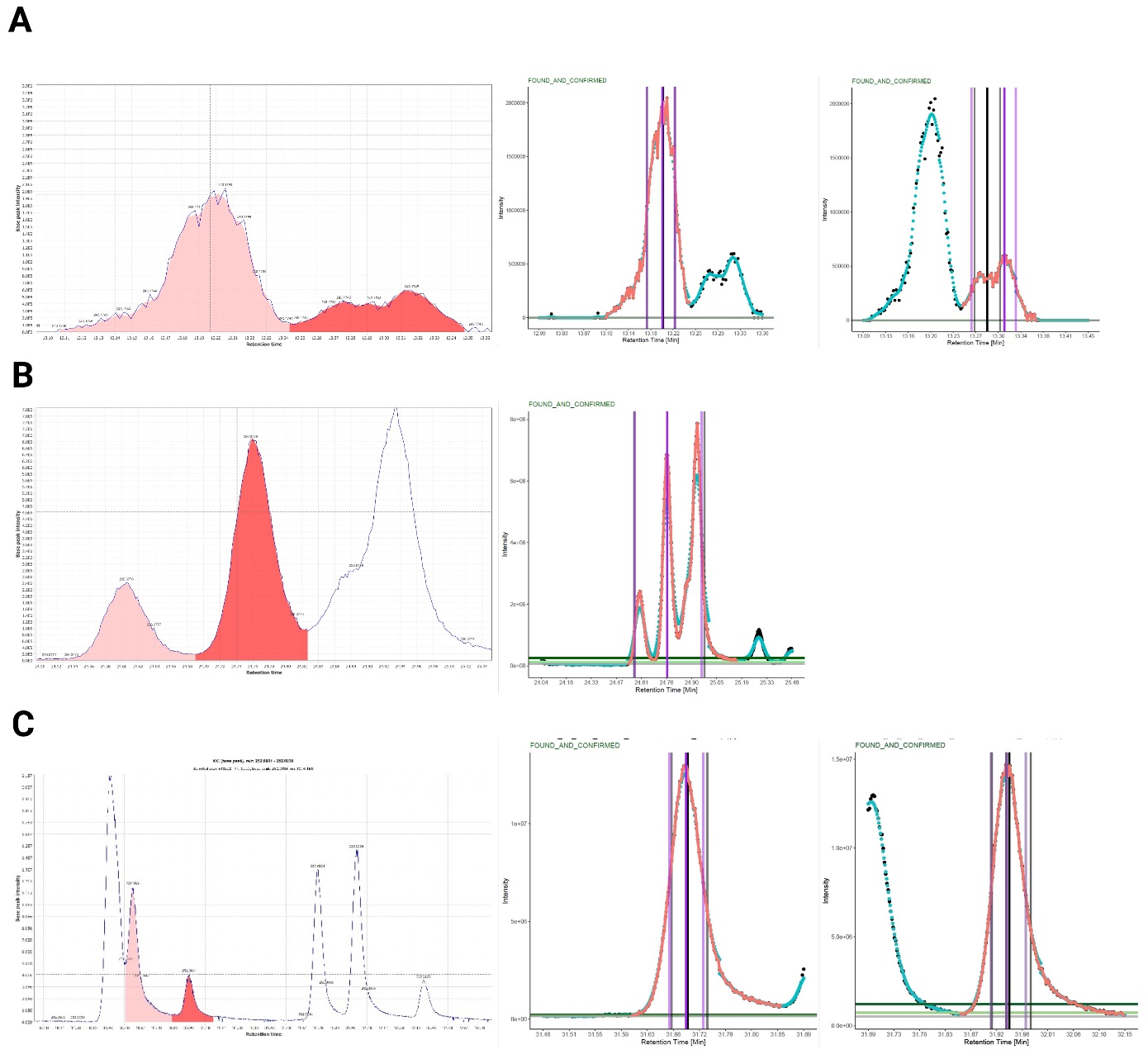


Figure S1-3: Examples of cases where ATS is outperforming MZmine.

A) EICs of tonalide and galaxolide. The left EIC with two red peaks shows how MZmine is able to split both peaks, but lists both in the feature list as galaxolide and tonalide. ATS is able to split the peaks and automatically assigns the correct peaks (left galaxolide, right tonalide) throughout the samples, even with shift-correction.

B) EICs of Cyclopenta(cd)pyren−3,4−H−one. The left EIC shows the handling of MZmine which splits the three peaks and assigns the compound individually. ATS merges and integrates all three peaks throughout the samples.

C) EICs for Benzo[a]pyrene and Benzo[e]pyrene. Left is the EIC from MZmine without shift-correction, showing that the algorithm will integrate two peaks which are however at the wrong retention time (correct would be the fourth and fifth peak starting on the left). ATS also detects both peaks but also accounts for the shift and correctly integrates the respective peaks. EIC = extracted ion chromatograms. Vertical lines in ATS indicate retention times of interest (black = expected apex retention time, purple = measured start, end, and apex retention times).

**Text S7. Examples of problematic compounds for ATS.**

Examples of false negative and false positive cases were selected and illustrated using TraceFinder and ATS in Figure S1-4.


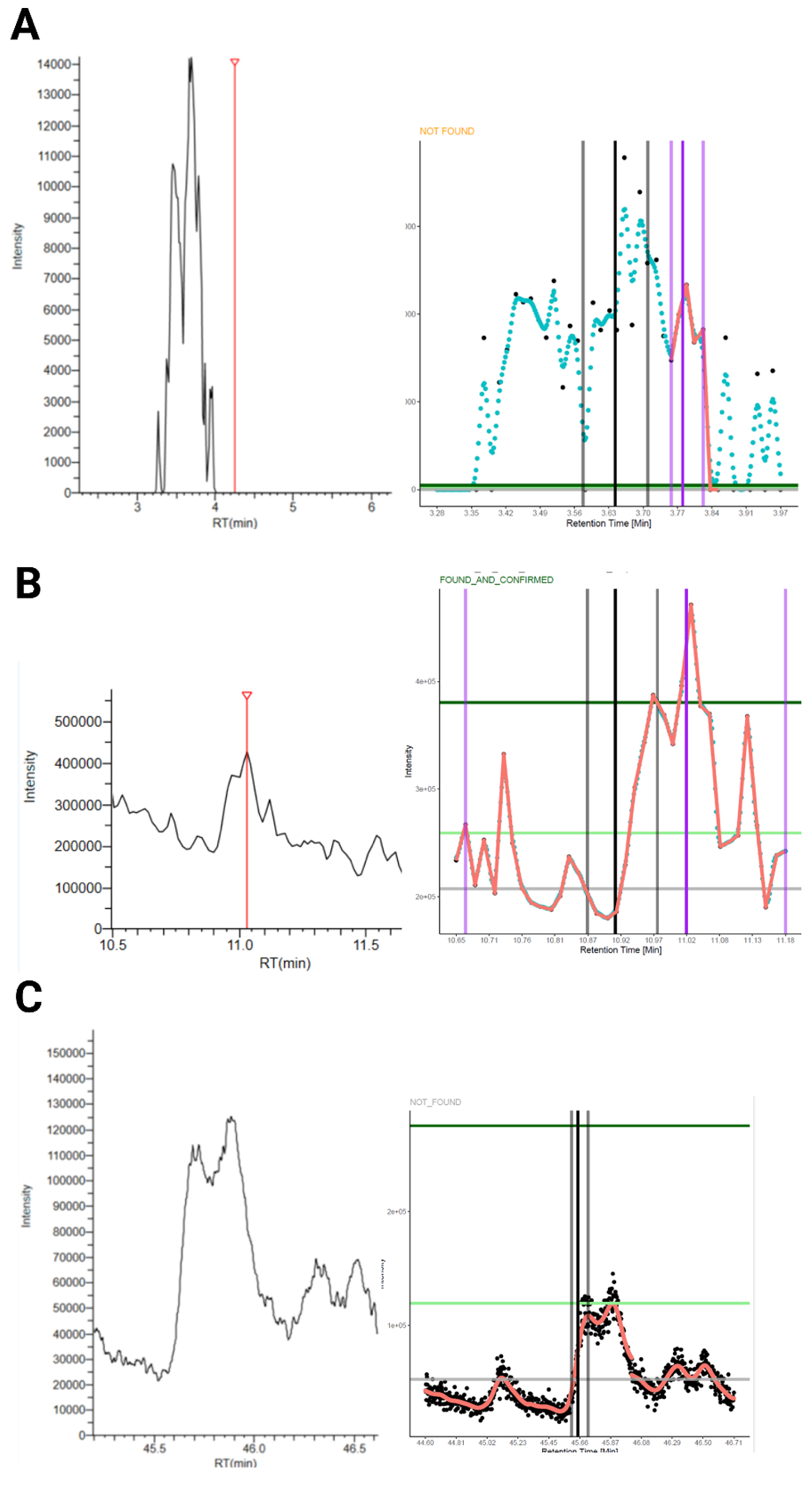


Figure S1-4: Examples of problematic compounds in ATS.

Extracted ion chromatograms generated in TraceFinder (left) and ATS (right). A) p-Toluenesulfonamide B) Naproxen C) Dibenzo(ae)pyrene. For ATS: Grey horizontal line = baseline, light-green horizontal line = in-sample limit of detection, dark-green horizontal line = in-sample limit of quantitation, black vertical line = expected retention time, grey vertical lines = expected start and end retention times at 50% peak intensity, dark-purple vertical line = detected retention time, light-purple lines = detected start and end retention times at 50% peak intensity.

**Text S8. Runtime of ATS.**

The runtime of ATS for all the samples was compared and summarized in Table S1-4.

Table S1-4: Runtime comparison of ATS for all samples

| Sample | Nr of analytes | Nr of files | Elapsed time (min) | Time per analyte (min) | Time per file (min) |
| --- | --- | --- | --- | --- | --- |
| LC_ESIpos_raw | 557 | 35 | 101.23 | 0.18 | 2.89 |
| LC_ESIpos_mzML | 557 | 35 | 122.26 | 0.22 | 3.49 |
| LC_ESIneg_raw | 150 | 35 | 43.58 | 0.29 | 1.25 |
| LC_ESIneg_mzML | 150 | 35 | 55.40 | 0.37 | 1.58 |
| GC_EI_raw | 188 | 37 | 99.19 | 0.53 | 2.68 |
| GC_EI_mzML | 188 | 37 | 76.16 | 0.41 | 2.05 |

# References

1. Niu L, Henneberger L, Huchthausen J, Krauss M, Ogefere A, Escher BI. pH-dependent partitioning of ionizable organic chemicals between the silicone polymer polydimethylsiloxane (PDMS) and water. ACS Environ Au. 2022;2(3):253-262.

2. Lin J, Keogh E, Wei L, Lonardi S. Experiencing SAX: a novel symbolic representation of time series. Data Min Knowl Disc. 2007;15:107-144.

3. Müller E, Huber CE, Brack W, Krauss M, Schulze T. Symbolic Aggregate Approximation Improves Gap Filling in High-Resolution Mass Spectrometry Data Processing. Anal Chem. 2020;92(15):10425-10432.
